# Prevention of Disease by Municipal Employment

**Published:** 1896-12

**Authors:** 


**A Monthly Review of Medicine and Surgery.**

*EDITORS:*

THOMAS LOTHROP, M. D. - - WM. WARREN POTTER, M. D.

All communications, whether of a literary or business nature, should be addressed to the managing editor: 284 FRANKLIN STREET, BUFFALO, N. Y.

VOL. XXXVI. DECEMBER, 1896. No. 5.

### PREVENTION OF DISEASE BY MUNICIPAL EMPLOYMENT.

WITH the approach of winter certain problems are always presented in a great city that are intimately related to the prevention of disease through the proper exercise of public and private charity and the employment of idle hands. These questions are always boldly accentuated at this season of the year in Buffalo, when after the close of navigation so many lake and canal workers are discharged from service. An increase of the sick-rate follows steadily in the track of idleness.

At a period when every avenue is exhausted to improve vital statistics, and especially when Buffalo has been so conspicuous through the marvelous reduction of her death-rate, it becomes her municipal officers to exercise watchful care lest through some neglect this record fails to be maintained. In addition to the ordinary demands upon private charity, which liberal people meet with great promptitude, the question is now presented to municipal officers as to the proper method of dealing with a large class of idle laborers that are annually foisted upon us by the discharge from service of those who can usually obtain employment in summer, but who in winter are compelled to makeshift as best they can. If these men and women, together with the families dependent upon them, can be kept from hunger and cold they, generally speaking, will keep healthy and strong. If not, they will grow feeble, become sick, and in many instances die after prolonged suffering, which latter is often more painful to witness than death itself.

In order to meet the exigencies of the situation, shelter, fuel, food and clothing must be provided in ample quantities to prevent suffering and to promote health. How can this best be done ? An intelligent answer to this question will present a solution of the whole problem. To support these people in idleness would be the very worst form of charity that could be offered. If a fund could be created sufficient to provide for their daily necessities with the certainty of leaving them unemployed, it would be a sad exercise of a cardinal virtue. Employment is what these people need, and the only adequate way to meet the situation is to provide it promptly when needed.

The question, then, becomes one of method rather than means. How shall the idle be employed ? How *can* they be employed ? is the real question. Obviously, to depend upon the imperfect and slow methods of private employment would not meet an emergency. Hundreds of men must be set at work, and that instantly, when the crisis comes. Hence, the only feasible way out is for the municipal authorities to come to the rescue. Fortunately, in Buffalo there is ample opportunity, generally speaking, for the employment of a large force of men to advantage and with economy. The necessity for the work will surely arise, and the men not otherwise engaged will be found eager to do it.

The excellent quality of our streets and their cleanliness in summer is our boast and pride. We are justly famous in this regard, and are the envy of visitors from other cities. Fortunately, these visitors generally come to us in summer, when our 240 miles of smooth pavements on broad streets and shaded avenues, kept almost scrupulously clean, show to the best advantage, eliciting the merited admiration and praise of our guests. If, however, they should visit us in winter we fear they would not be prompted to unstinted commendation of the condition of our streets. An unlimited supply of snow and ice is bad enough for stone pavements, but when they accumulate on the asphalted streets the condition becomes almost unendurable.

The business traffic on Main street has become so enormous that due regard for safety of life and limb, as well as economy in horses and carriages, demand that every precaution be taken to keep it clear of all obstructions, no matter whether of a natural or artificial kind. This means, in our view, that snow should be removed as fast as it falls between the foot of Main street and High street, and on the cross-streets far enough east and west of Main to make traffic comfortable as well as safe. This should be done so well as to prevent absolutely the accumulation of ice within the limits named. Such scenes as are witnessed every spring on some portions of Main street, as well as on some of the cross-streets, of which Chippewa may be cited as an example, in the cleaving of ice that is from one to three feet in thickness, piling it in great heaps and allowing it to melt in slow and uncertain processes, or to be carted away at the pleasure of the street commissioner, ought never to be repeated in a city with the conditions and opportunities that all concede belong to Buffalo. These provincial methods, pertaining to a period long past, ought not still to prevail in this city if we would hold it up as a model in municipal administration as well as an ideal for business or residence purposes.

New York, formerly one of the worst governed cities, is rapidly becoming the best, and its street cleaning department is an example of excellence that may well challenge admiration and serve as a model for other cities. With its street cleaning brigades, clad in their white sanitary uniforms, attacking the snow in the business parts of the city and clearing it away before noon on snowy days, a spectacle is furnished that becomes an object lesson in street sanitary police regulations.

But let us return to our purpose. If the street department of Buffalo were authorised to employ an adequate number of idle men to keep the business part of the city entirely free from snow and ice during the approaching winter, we are quite sure that it would receive the approbation of the entire people. Business men will praise it because the approaches to their stores, shops and factories have been accessible at all times without inconvenience or danger. Owners of horses and carriages who drive or ride for either business or pleasure will bless it for its humanity to animals, to say nothing of the saving in wear and tear of vehicles. The poor and unemployed will certainly send up a prayer of approval, while philanthropists, sanitarians and progressive people everywhere in the city will say, "Well done, good and faithful servants," to all concerned in consummating a reform in the economics of municipal authority so devoutly to be wished. The municipal government should make haste to avail itself of such a splendid opportunity to contribute its influence and power toward the prevention of disease.

